# Health system measurement: Harnessing machine learning to advance global health

**DOI:** 10.1371/journal.pone.0204958

**Published:** 2018-10-05

**Authors:** Hannah H. Leslie, Xin Zhou, Donna Spiegelman, Margaret E. Kruk

**Affiliations:** 1 Department of Global Health and Population, Harvard T.H. Chan School of Public Health, Boston, Massachusetts, United States of America; 2 Department of Epidemiology, Harvard T.H. Chan School of Public Health, Boston, Massachusetts, United States of America; 3 Department of Biostatistics, Harvard T.H. Chan School of Public Health, Boston, Massachusetts, United States of America; 4 Department of Nutrition, Harvard T.H. Chan School of Public Health, Boston, Massachusetts, United States of America; 5 Center on Methods for Implementation and Prevention Science, Yale School of Public Health, New Haven, Connecticut, United States of America; The University of Warwick, UNITED KINGDOM

## Abstract

**Background:**

Further improvements in population health in low- and middle-income countries demand high-quality care to address an increasingly complex burden of disease. Health facility surveys provide an important but costly source of information on readiness to provide care. To improve the efficiency of health system measurement, we applied unsupervised machine learning methods to assess the performance of the service readiness index (SRI) defined by the World Health Organization and compared it to empirically derived indices.

**Methods:**

We drew data from nationally representative Service Provision Assessment surveys conducted in 10 countries between 2007 and 2015. We extracted 649 items in domains such as infrastructure, medication, and management to calculate an index using all available information and classified facilities into quintiles. We compared three approaches against the full item set: the SRI, a new index based on sequential backward selection, and an enriched SRI that added empirically selected items to the SRI. We evaluated index performance with a cross-validated kappa statistic comparing classification using the candidate index against the 649-item index.

**Results:**

9238 facilities were assessed. The 49-item SRI performed poorly against the index using all 649 items, with a kappa value of 0.35. New empirically derived indices with 50 and 100 items captured much more information, with cross-validated kappa statistics of 0.71 and 0.80, respectively. Items varied across the indices and in sensitivity analyses. A 100-item enriched SRI reliably captured the information from the full index: 83% of the facilities were classified into correct quintiles of service readiness based on the full index.

**Conclusion:**

A facility readiness measure developed by global health experts performed poorly in capturing the totality of readiness information collected during facility surveys. Using a machine learning approach with sequential selection and cross-validation to identify the most informative items dramatically improved performance. Such approaches can make assessment of health facility readiness more efficient. Further improvements in measurement will require identification of external criteria—such as patient outcomes—to guide and validate measure development.

## Introduction

The current era in global health is marked by pursuit of the Sustainable Development Goals (SDGs) for 2015–2030. In contrast to the Millennium Development Goals, the SDGs include an explicit commitment to universal health coverage and a recognition of the increasingly varied global disease burden, including chronic conditions such as diabetes that require continuous and coordinated health services.[1] High-quality health systems will be necessary to deliver this care and achieve the ambitious health-related SDGs.[2, 3] Whether health systems in low- and middle-income countries (LMIC) have the capacity to provide quality care is the subject of increasing scrutiny.[3–7]

While national health information systems remain under development in many LMIC,[8] periodic health facility surveys can provide valuable information on health system capacity.[9, 10] These assessments can cover hundreds of individual items, from medications to diagnostic tests. The World Health Organization (WHO) has defined the items required to demonstrate basic readiness to provide health services and defined measures to reduce the information from health facility assessments into facility- or service-level indices.[9] For instance, the general service readiness index (SRI) includes 50 items in the domains of basic amenities (infrastructure), infection control, equipment, diagnostics, and medication; it is intended to capture the essential foundation needed to provide basic health services.

Although research using health facility assessments is increasing,[11–15] there is little evidence that information from facility surveys is used to inform national policy on service allocation or health system strengthening.[8] The impact of investments in conducting such surveys—from $100,000 for a small survey to much more for nationally representative system assessments in populous countries[9]–is undermined by the limited use of the resulting data. While the SRI is the most commonly used summary measure for such facility assessments, it uses a fraction of the total information collected. Health policy makers may be reluctant to make use of information from health facility assessments without an indication of the value of such measures in distinguishing better and worse equipped facilities or in representing the overall capacity of a facility. In high-income countries, machine learning approaches have been applied to routine health information data to extract insight from large datasets.[16, 17] These approaches employ predictive algorithms that learn from the data without overfitting, with the goal of reducing large, unwieldy data to useful and usable summaries.[18] Application of these methods is limited in low-resource contexts to date, despite their potential utility in deriving insight from data.

The objective of this study is to develop summary measures of health facility capacity using machine learning approaches of sequential selection and cross-validation in order to enhance efficiency of and insights provided by existing health facility assessments. We assess the performance of the SRI in capturing full facility readiness and test new measures to summarize readiness with fewer items.

## Materials and methods

### Ethical approval

The original survey implementers obtained ethical approvals for data collection; the Harvard University Human Research Protection Program deemed this analysis exempt from human subjects review.

### Study sample

We identified the Service Provision Assessment (SPA) surveys as the most detailed nationally representative health system assessments and included all assessments conducted between 2006 and 2015 (pre-2006 assessments focused on either HIV or maternal and child health alone). SPA surveys were conducted in ten countries in this decade: Bangladesh, Haiti, Kenya, Malawi, Namibia, Nepal, Rwanda, Senegal, Tanzania, and Uganda, with repeat surveys in Tanzania (2006 and 2015) and three annual surveys in Senegal (2013, 2014, 2015). In most countries, the SPA draws a representative sample of both public and private health facilities, with stratified sampling in urban and rural locations and oversampling of hospitals. Haiti, Malawi, Namibia, and Rwanda conducted a census or near census of all health facilities; Bangladesh did not sample small private facilities. Facilities assessed in both the 2013 and 2015 waves of the Senegal SPA were dropped from the 2013 data to eliminate duplicate observations. We also excluded health huts, extension facilities in Senegal that were assessed using an abbreviated survey.

Each survey entailed a facility audit consisting of interviews with facility and service managers and direct verification of the resources available for care, including management, staff, supplies, equipment, medication, and diagnostics. Areas assessed include facility-wide resources and services such as central pharmacy and laboratory as well as specific clinical services such as HIV, delivery care, and child health; common items such as infection prevention measures are repeated across multiple services. The assessment tool was modified in 2012 to include basic readiness for non-communicable disease care and minor surgery in addition to its prior focus on maternal health, child health, and infectious diseases.

### Facility readiness indices

We defined two summary measures for each facility: SRI based on the 2013 definition from WHO[19] and an index based on all available items of facility readiness in each survey. All items are binary, with 1 indicating the item was observed present (and functional as applicable) and 0 indicating the item was not present or could not be assessed, e.g. due to the relevant service not being offered. Due to the evolution of the survey and country-to-country variation, between 37 and 49 of the 50 items in the SRI definition could be extracted for each country. Items fell into 5 domains: infrastructure (7 items), equipment (6 items), infection prevention (9 items), diagnostic capacity (8 items), and medication (20 items). Items were averaged within domain; domain scores were averaged to provide the final index, ranging from 0 to 1. Following the logic of the SRI, we extracted all possible items in these five sub-domains as well as an additional domain of human resources and management in order to capture all inputs to care assessed in the SPA, a total of 649 items. Items were averaged within domain, and the overall index was calculated as the average readiness across these six domains (0 to 1).

### Analysis

We report descriptive statistics using the survey sampling weights. We classified facilities into quintiles using the 649-item index to identify better and worse performing facilities in terms of overall readiness. We classified facilities into quintiles using the full measure and the original SRI and compared classifications using the kappa statistic, a measure of inter-rater reliability.

In this analysis, no external information was available to serve as a source of validation for facility readiness; we undertook unsupervised machine learning using the information from within the dataset—namely the 649-item index—as the reference criterion. The development of new readiness indices using this machine learning approach involved two steps: selection and evaluation. We first implemented sequential backward and sequential forward selection of individual items.[20] Sequential backward selection entailed discarding one item of the 649, recalculating the index and reclassifying facilities into quintiles, and calculating a kappa statistic to assess performance against the original measure. This procedure was applied for each of the 649 items, with the item whose exclusion resulted in the least loss of reliability (highest kappa statistic) dropped and the procedure repeated on the remaining items. Sequential forward selection is similar but started with an empty set and tested all possible single-item measures before retaining the best performing item based on the kappa statistic and repeating. We compared backward and forward selection based on the magnitude of the kappa statistic for a given number of items.

To evaluate the performance of an index of a given number of items, *M*, we used a 10-fold cross-validation procedure. The data were randomly partitioned into 10 roughly equal-sized parts. Nine parts were taken as training data and used to choose the *M* items using selection as described above. To obtain the cross-validated kappa statistic for each *M*-item score, we chose the best *M* items in the training data and calculated the new index based on those items in the tenth part. We repeated item selection in each training set and calculated the resulting index in the validation fold until all facilities had an *M*-item index determined by the other 9 folds. Indices were based on the same number of items for each fold, but the specific items may have differed between folds, at least in part, as selected by the training data. We then classified facilities into quintiles by their *M*-item index and computed a kappa statistic to assess performance of the *M*-item index against the original 649-item measure. This statistic provided an estimate of expected performance of an index with a given number of items. The procedure was conducted for all possible numbers of items, from 1 to 648. Following cross-validation, we chose the items for each *M*-item index using the full data set and selecting items 1 to *M* by order of selection.

We first developed new indices with no pre-specified items and plotted performance using the cross-validated kappa statistic for indices of 648 to 1 item. As a second approach, we developed an enriched SRI with empirically selected items added to the existing SRI and assessed the performance of this enriched metric from 648 items to 50 items. As sensitivity analyses, we repeated the analysis within subsets of facilities (hospitals and non-hospitals) and by tertiles and deciles instead of quintiles.

We selected empirically defined indices of 50 and 100 items (equivalent to or twice as long as the original SRI, respectively) and an enriched SRI index of 100 items as candidate shorter measures. We classified these indices into quintiles and compared this classification to quintiles of readiness based on the full 649-item index using percent agreement and a kappa statistic calculated on the full sample.

## Results

A total of 9,976 facilities were selected for assessment from master facility lists; 9,690 assessments were successfully conducted (97.1% response). We excluded 452 assessments in Senegal from the analysis (191 that were repeated surveys and 261 health huts) for an analytic sample of 9,238 health facilities in ten countries (Table 1). The average SRI ranged from 0.42 in Bangladesh and Uganda to 0.70 in Namibia. Readiness based upon all 649 items was consistently less than the SRI: average readiness exceeded 0.50 in Namibia alone and fell below 0.40 in most countries.

**Table 1:** Sample characteristics (N = 9238).

Country	Survey year	Facilities in analytic sample	SRI (mean ± SD)	649-item index (mean ± SD)
Bangladesh	2014	1548	0.42 ± 0.14	0.26 ± 0.11
Haiti	2013	905	0.55 ± 0.16	0.35 ± 0.12
Kenya	2010	695	0.56 ± 0.17	0.39 ± 0.15
Malawi	2013	977	0.58 ± 0.15	0.40 ± 0.14
Namibia	2009	411	0.70 ± 0.13	0.52 ± 0.13
Nepal	2015	963	0.47 ± 0.13	0.32 ± 0.10
Rwanda	2007	538	0.60 ± 0.15	0.46 ± 0.14
Senegal^1^	2013	173	0.57 ± 0.09	0.39 ± 0.08
Senegal	2014	363	0.63 ± 0.10	0.44 ± 0.12
Senegal	2015	375	0.65 ± 0.09	0.46 ± 0.12
Tanzania	2006	611	0.45 ± 0.13	0.36 ± 0.10
Tanzania	2015	1188	0.50 ± 0.17	0.39 ± 0.12
Uganda	2007	491	0.42 ± 0.16	0.34 ± 0.14

^1^ Excluding facilities also assessed in 2015. SD: Standard deviation. SRI: Service readiness index

The kappa statistic for SRI and the full index was 0.35, indicating these indices agreed on facility classification 35% of the time beyond chance alone. This kappa value suggests minimal agreement.[21] This lack of agreement is further illustrated in the first panel of Table 2: SRI as defined by the WHO classifies facilities divergently from the full index, with only 4,445 facilities (48%) classified in the same quintiles. While no facilities in the best group for one index were in the worst for the other, 275 facilities in the top two quintiles of all facilities based on the SRI were in the bottom two quintiles using the full index.

**Table 2:** Classification of health facilities into quintiles of readiness (N = 9238).

A: Full index vs. SRI*							
			WHO SRI				
		N Mean readiness	Quintile 1 (Best)	Quintile 2	Quintile 3	Quintile 4	Quintile 5 (Worst)
Full index	Quintile 1	1848 0.64	67.9%	26.4%	5.2%	0.5%	0.0%
	Quintile 2	1848 0.50	22.5%	38.1%	31.1%	7.6%	0.7%
	Quintile 3	1847 0.41	7.9%	22.7%	32.3%	27.9%	9.2%
	Quintile 4	1848 0.31	1.9%	11.0%	22.6%	38.4%	26.1%
	Quintile 5	1847 0.20	0.0%	1.9%	8.5%	25.6%	64.0%
B: Full index vs. 50-item empirical index*							
			50-item empirical index				
		N Mean readiness	Quintile 1 (Best)	Quintile 2	Quintile 3	Quintile 4	Quintile 5 (Worst)
Full index	Quintile 1	1848 0.64	88.4%	11.6%	0.0%	0.0%	0.0%
	Quintile 2	1848 0.50	11.6%	74.7%	13.6%	0.1%	0.0%
	Quintile 3	1847 0.41	0.1%	13.6%	72.1%	14.2%	0.0%
	Quintile 4	1848 0.31	0.0%	0.2%	14.1%	76.0%	9.7%
	Quintile 5	1847 0.20	0.0%	0.0%	0.0%	10.0%	90.0%
C: Full index vs. 100-item empirical index*							
			100-item empirical index				
		N Mean readiness	Quintile 1 (Best)	Quintile 2	Quintile 3	Quintile 4	Quintile 5 (Worst)
Full index	Quintile 1	1848 0.64	91.9%	8.1%	0.0%	0.0%	0.0%
	Quintile 2	1848 0.50	8.1%	82.8%	9.0%	0.0%	0.0%
	Quintile 3	1847 0.41	0.0%	9.0%	82.1%	8.9%	0.0%
	Quintile 4	1848 0.31	0.0%	0.0%	8.9%	85.0%	6.1%
	Quintile 5	1847 0.20	0.0%	0.0%	0.0%	6.1%	93.9%
D: Full index vs. 100-item enriched SRI*							
			100-item enriched SRI				
		N Mean readiness	Quintile 1 (Best)	Quintile 2	Quintile 3	Quintile 4	Quintile 5 (Worst)
Full index	Quintile 1	1848 0.64	89.7%	10.3%	0.0%	0.0%	0.0%
	Quintile 2	1848 0.50	10.3%	78.8%	10.9%	0.0%	0.0%
	Quintile 3	1847 0.41	0.0%	11.0%	77.2%	11.9%	0.0%
	Quintile 4	1848 0.31	0.0%	0.0%	11.9%	78.6%	9.5%
	Quintile 5	1847 0.20	0.0%	0.0%	0.0%	9.5%	90.5%

SRI: Service readiness index

**Fig 1:**
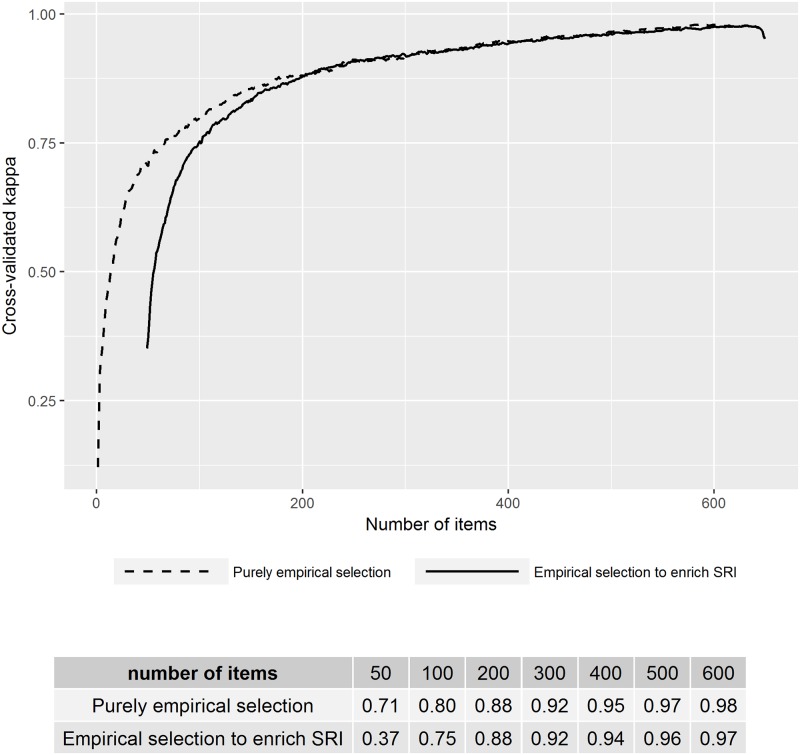
Cross-validated kappa statistic for indices created using sequential backward selection.

* The kappa statistics for the comparisons are 0.35, 0.75, 0.84, and 0.79 respectively; these are based on the full sample, in contrast to the cross-validated kappa statistics reported in Fig 1.

Sequential backward selection required more computing time (4.6 vs. 2.3 hours) but outperformed sequential forward selection in all analyses based on the cross-validated kappa statistic at any given number of items (S1 and S2 Figs); we thus present results from sequential backward selection only. Indices selected using sequential backward selection with no pre-specified items performed very well against the full index, particularly when large numbers of items were retained: cross-validated kappa exceeded 0.88 for at least 200 items and declined to 0.80 for 100 items and 0.71 for 50 items (Fig 1). The performance of the 50- and 100-item indices—measures that could provide considerable efficiency by cutting the facility assessment to under 20% of its current length—is detailed in Table 2 Panels B and C. These empirical indices outperform the original SRI compared to quintiles based on the full index, with 80% of facilities (7,412) classified correctly by the 50-item empirical index and 87% of facilities (8,051) classified correctly by the 100-item empirical index. With 100 items, no facility is misclassified by more than one quintile.

The content of the 100-item empirical index is shown in S1 Table; it included only 8 SRI items. The selected items reflect the breadth of the facility assessment rather than a coherent picture of facility readiness; for example, 11 of 16 amenities items pertain to client privacy, while four different diagnostic items address the availability of rapid HIV tests in distinct areas of the facility. Sensitivity analyses limiting the sample to hospitals or non-hospitals or assessing the performance relative to the full index using tertiles or deciles returned comparable results in terms of improving on the original SRI, but with substantial differences in the list of items selected (results not shown).

The second approach attempted to enrich the SRI. The enriched SRI performed comparably to the empirical indices with large numbers of items, with reliability declining more sharply below 150 items (Fig 1). The cross-validated kappa statistic for a 100-item enriched SRI was 0.75 (compared to 0.80 for the 100-item empirical index). As shown in Table 2 Panel D, 83.0% of the facilities (7,663) were correctly classified into quintiles by the 100-item enriched SRI, with no facilities misclassified by more than one quintile. Below 100 items, reliability declined substantially to 0.37 for 50 items (the original SRI plus 1 item).

A comparison of the 100-item enriched SRI and original SRI shows the substantial improvements contributed by the additional items: 49% facilities disagree by at least one quintile, including 8% by more, with a kappa statistic of 0.39 (S2 Table).

Table 3 lists the items retained in the 100-item enriched SRI, sorted by domain and, for those not included in the original SRI, their selection order. These 100 items are relatively evenly distributed across the six domains, ranging from 13 equipment items (6 in the original SRI) to 24 medications (19 in original SRI). Selected items include both facility-wide attributes such as a daily update of medication availability and service-specific items such as privacy in the family planning exam room or syringes in sick child rooms. Thirty-five of 100 items are present in both the enriched SRI and purely empirical indices.

**Table 3:** Contents of 100-item enriched SRI.

Domain	Items	Item is in SRI	Selection order^1^
Amenities	At least one room with visual and auditory privacy	1	
	Improved water source available year-round within 500 meters	1	
	Computer and internet	1	
	Central electricity or functional generator with fuel	1	
	Facility phone or short-wave radio available at all times	1	
	At least one functional client toilet observed (if observed an option)	1	
	Functional ambulance with fuel	1	
	Delivery area has visual and auditory privacy	0	3
	Family planning exam area has visual and auditory privacy	0	7
	PMTCT area private room	0	10
	ANC exam area private room	0	18
	Vaccination area has visual and auditory privacy	0	26
	NCD exam area private room	0	32
	Phlebotomy area private room	0	35
Equipment	At least one adult scale	1	
	At least one pediatric scale	1	
	At least one stethoscope	1	
	At least one thermometer	1	
	At least one exam light	1	
	At least one blood pressure apparatus	1	
	Needle holder observed in delivery area	0	5
	Skin disinfectant (other than chlorhexidine) in delivery area	0	12
	Functional height or length board in child area	0	16
	Pediatric self-inflating bag and mask available and functioning in outpatient area	0	22
	Suture material with needle observed in delivery area	0	33
	Filled Oxygen cylinder available and functioning in outpatient area	0	34
	Antiseptic solution for IUCD or implant methods	0	42
Infection control	At least one room with sharps disposal box	1	
	Medical or contaminated waste is adequately disposed of	1	
	At least one room with waste bin with pedal and liner	1	
	Sharps are adequately disposed of	1	
	At least one room with surface disinfectant	1	
	At least one room with gloves	1	
	At least one room with single-use syringe	1	
	At least one room with soap & water or hand disinfectant	1	
	At least one guideline for standard precaution / infection control observed	1	
	Delivery room: sharps box observed	0	6
	TB exam area: soap observed	0	14
	Child vaccination area: face mask observed	0	19
	Sick child room: single-use syringe observed	0	28
	ANC exam room: soap observed	0	31
	ANC exam room: hand disinfectant observed	0	37
	NCD exam area: face mask observed	0	41
	Outpatient exam area: apron / gown observed	0	46
	TB exam area: sharps box observed	0	47
	Blood draw area: gloves observed	0	49
Diagnostics	Diagnostics: functional syphilis testing	1	
	Diagnostics: functional urine dipstick test for glucose	1	
	Diagnostics: functional hemoglobin / anemia testing	1	
	Diagnostics: functional HIV testing	1	
	Diagnostics: functional urine dipstick test for pregnancy	1	
	Diagnostics: functional malaria testing	1	
	Diagnostics: functional blood glucose testing	1	
	Diagnostics: functional urine dipstick test for protein	1	
	HIV rapid diagnostic test observed and valid in HIV area	0	9
	HIV rapid diagnostic test observed and valid in ANC area	0	15
	Diagnostics: TB test with light or fluorescent microscope, slides & ZN stain	0	17
	Urine glucose test observed and valid in ANC area	0	23
	HIV rapid diagnostic test observed and valid in TB area	0	29
	Filter paper cards for dried blood spot collection observed and valid in PMTCT area	0	38
	HIV rapid diagnostic test observed and valid in STI area	0	45
	Syphilis rapid diagnostic test observed and valid in ANC area	0	48
Medication	Ceftriaxone injection observed and valid	1	
	ORS in pharmacy or sick child area	1	
	Ampicillin powder for injection observed and valid	1	
	Amoxicillin or ampicillin tablet observed & valid	1	
	Beclomeasone inhaler observed and valid	1	
	Ibuprofen [brufen] observed and valid	1	
	Enalapril tablet or alternative ACE inhibitor observed and valid	1	
	Omeprazole or alternative -prazole tab observed and valid	1	
	Paracetamol tablets observed and valid	1	
	Zinc tablet or syrup observed and valid	1	
	Metformin tablets observed and valid	1	
	Amitriptyline tablets observed and valid	1	
	Simvastatin or other statin observed and valid	1	
	Gentamicin injection observed and valid	1	
	Amoxicillin syrup observed and valid	1	
	Insulin injection observed and valid	1	
	Salbutamol inhaler observed and valid	1	
	Glibenclamide tablets observed and valid	1	
	Amlodipine tablet or alternative Ca channel blocker observed and valid	1	
	Tetanus toxoid vaccine available and valid in antenatal care area	0	11
	Combined estrogen-progesterone oral contraceptive pills observed in FP area	0	21
	Isoniazid + rifampicin + pyrazinamide + ethambutol (4fdc) observed and valid	0	39
	DPT+Hib+HepB vaccine observed, in stock, and valid	0	40
	Valid injectable uterotonic (oxytocin / ergometrine) available	0	44
Management	Computer or stock ledger updated daily with antiretroviral medicines available	0	1
	Health records for family planning clients maintained on site (observed)	0	2
	Test referral record observed in HIV or TB service	0	4
	Staff community meeting within 6 months	0	8
	Observed report of health services info compiled at least every 4–6 months	0	13
	Health records for antenatal care clients maintained on site (observed)	0	20
	Supervisor used checklist for quality of health services data	0	24
	Routinely carries out quality assurance activities	0	25
	Record of management team meeting observed	0	27
	Supervisor discussed problems	0	30
	Computer or stock ledger updated daily with medicine available	0	36
	Medications, vaccines and contraceptives stored according to expiry date	0	43
	Health records for sick children maintained on site (observed)	0	50
	Supervisor discussed facility performance based on data	0	51

SRI: Service readiness index.

^1^ Selection order based on backward selection on full sample with 49 SRI items predetermined: items shown are numbered 1 to 51 where item 1 was the last item discarded in the backwards selection procedure (most informative item conditional on pre-selection of the SRI items).

## Discussion

This study of over 9,000 health facilities in ten countries is the first effort to apply machine learning to derive insight and improve efficiency of health facility survey data in LMICs. The results demonstrate that the SRI as defined by the WHO captures only a portion of the information contained in detailed facility assessments and may result in highly divergent classification of facilities as poorly or well prepared to provide high-quality care. Purely empirical indices captured much of the information of the full survey with many fewer items, although the items selected varied across sensitivity analyses. Enriching the SRI with additional items provided a blended approach between normative guidelines and empirical assessment. A 100-item index incorporating the SRI items proved reliable in capturing the full information contained in the facility surveys. This work demonstrates that the unsupervised machine learning approach applied—sequential backward selection with cross validation for evaluation[22]—provides a feasible approach to extract shorter measures from the data collected during health facility assessments as a step towards enhancing the use of these surveys. Further insights into health system performance will require better data for linking health facility inputs to patient perspectives and health outcomes.

Existing research on health facility readiness focuses primarily on describing overall readiness,[11, 12] identifying gaps in particular services,[23] and linking readiness to outcomes such as health service utilization.[24] These studies have identified deficiencies in SRI in multiple countries, from low-income nations like Malawi and Haiti to less poor countries like Kenya and Namibia,[11, 12] as well as low correlation between readiness and processes of care.[25] The findings of this work suggest that overall service readiness as measured by all input items in the SPA surveys is even lower in most health facilities than indices such as the SRI that focus on basic elements of readiness. This result adds to the growing recognition of deficiencies undermining the quality of facility infrastructure available in health systems in low- and middle-income countries.[3] In addition, the low concordance between rankings based on the SRI and those based on all available survey information demonstrates that the SRI is not a good proxy for readiness based on the full survey.

Can the SRI be improved? Using all readiness items in the SPA surveys as a guide, we developed considerably shorter indices that classified most facilities into quintiles correctly as compared to the full measure, with a cross-validated kappa statistic of 0.80 for the 100-item index. More efficient measurement is possible without losing much insight on facility readiness. However, the instability of this measure in terms of the items selected across sensitivity analyses and its lack of coherence suggests it may not be a compelling option for policy makers. This shortcoming may reflect the survey content as a whole: the breadth of items—including repeated assessment of common items such as privacy and infection control measures—and lack of predefined summary measures fit for purpose means that the reference point itself does not provide consistent insight on full facility readiness.

A blended approach combining the predefined SRI items with items added through empirical assessment provided shorter indices balancing normative coherence with concordance with the classification based on full information. The percent agreement for quintiles using the 100-item enriched SRI compared to the 649-item index was 83%, with a cross-validated kappa of 0.75, suggesting moderate inter-rater reliability.[21] The methods applied here can be refined to suit the needs of individual countries or analysts in terms of extracting insight from health facility data. The content of the enriched index highlight the range of items included in the SPA surveys, including both fixed infrastructure such as private rooms, major assets such as ambulances, and stocked items such as medication, supplies, and infection control measures that may fluctuate between available and out of stock on a regular basis. One limitation of measuring SRI in periodic surveys, no matter how well designed, is that information on physical stock is quickly out of date. Routine health information systems may be better placed to assess items that go out of date quickly such as medication stock, while periodic surveys may be best positioned to capture more costly but valuable measures such as facility function or performance.

Prior applications of machine learning analysis in health include questions such as predicting future disease or mortality using electronic health records[26, 27] or population based surveys.[17] While more limited, applications in health services research include efforts to improve risk adjustment for health insurance plan payments.[16] These studies have demonstrated improvements in synthesizing large and complex data into summary measures or predictions, using tools such as cross-validation. The current study confirms that such methods can be applied to derive insight from global health data as well.

One important limitation of the work is the lack of an external criterion, such as mortality, treatment success, or retention in care, to guide empirical selection. A supervised learning approach anchors the selection to a meaningful outcome and can identify reduced numbers of variables that are as or more predictive of this health outcome and presumably those related to it as full sets.[16] In the unsupervised analysis employed here, we are able to identify efficiencies in capturing the full set of items but not improve beyond what can be accomplished by this full set. Moving towards more efficient data collection might best require internal or external outcome data to validate the indices. Linking health system and population data in order to attribute population outcomes to the health system is a difficult undertaking in low-resource settings at the moment.[28] Without such external information, however, efforts to streamline data collection and enhance its utility are constrained. Increased coordination in data collection and country-led synthesis may be necessary to obtain linked health system quality and patient outcome data to enable more complex analysis of health system capacity and performance.

Other study limitations include inconsistencies in SPA surveys over time and between countries that prevented comparison of identical measures across countries. Survey questions were skipped if a service was not offered in a given facility; we set all such items to zero on the basis that the resources were not demonstrably present. Finally, the SPA surveys are cross-sectional and do not capture change over time or fluctuations in readiness; although these differences should not affect the main findings of this analysis, they limit the generalizability of the descriptive results to current health system readiness.

The findings of this analysis suggest that collecting an extensive number of items in each facility assessment is an inefficient use of resources and one that should be reconsidered as global and national stakeholders turn greater focus to health system capacity and performance. Moving forward, health system measurement should: 1) predefine the purpose of the data, including the form and purpose of the intended summary measures for synthesis and use of results, 2) optimize efficiency by blending expert opinion and empirical methods for the selection of items, and 3) include external items such as patient outcomes for validation. Better insight and informed action for health system strengthening are achievable and will prove to be important elements in improving the quality of care provided worldwide.

## Supporting information

S1 Fig Comparing sequential backward and sequential forward selection using the cross-validated kappa statistic: Purely empirical selection. (TIFF)

S2 Fig Comparing sequential backward and sequential forward selection using the cross-validated kappa statistic: Enriched SRI. (TIFF)

S1 Table Items in the 100-item empirical index, ordered by domain and selection order. ANC: Antenatal care. DPT: Diphtheria, pertussis, tetanus. Hib: Haemophilus influenzae type B. HIV: Human immunodeficiency virus. IUCD: Intrauterine contraceptive device. NCD: Non-communicable disease. SRI: Service readiness index. STI: Sexually transmitted infection. TB: Tuberculosis. ZN: Ziehl-Neelsen.
(DOCX)

S2 Table Classification of facilities using original SRI and 100-item enriched SRI. SRI: Service readiness index.
(DOCX)
