# Supplementary figures and images for: Health system measurement: Harnessing machine learning to advance global health
Source: PLoS One. 2018 Oct 5;13(10):e0204958. doi: 10.1371/journal.pone.0204958 (PMC6173424; doi:10.1371/journal.pone.0204958)

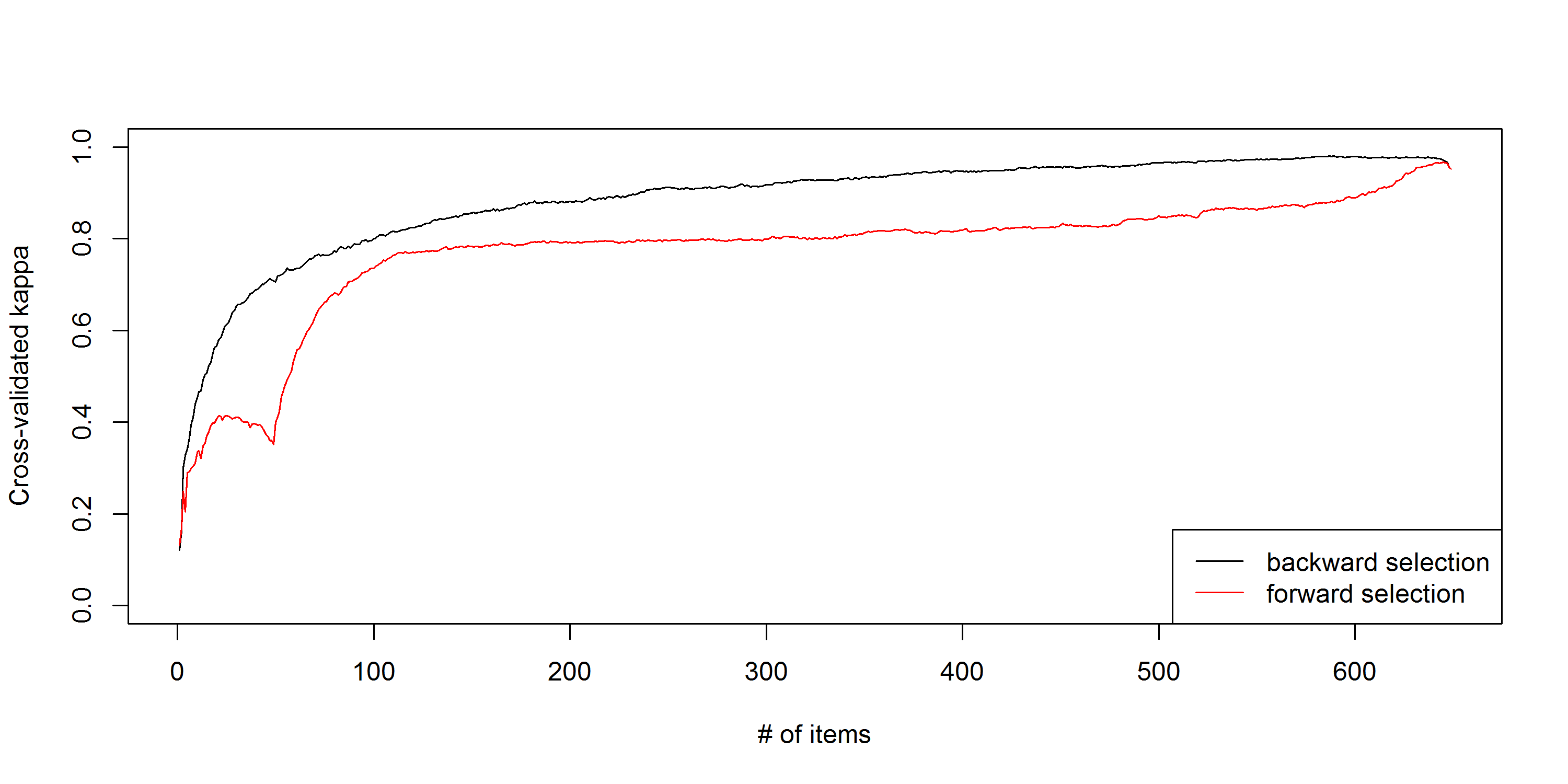

Supplement: S1 Fig — (TIFF) [file pone.0204958.s001.tiff]

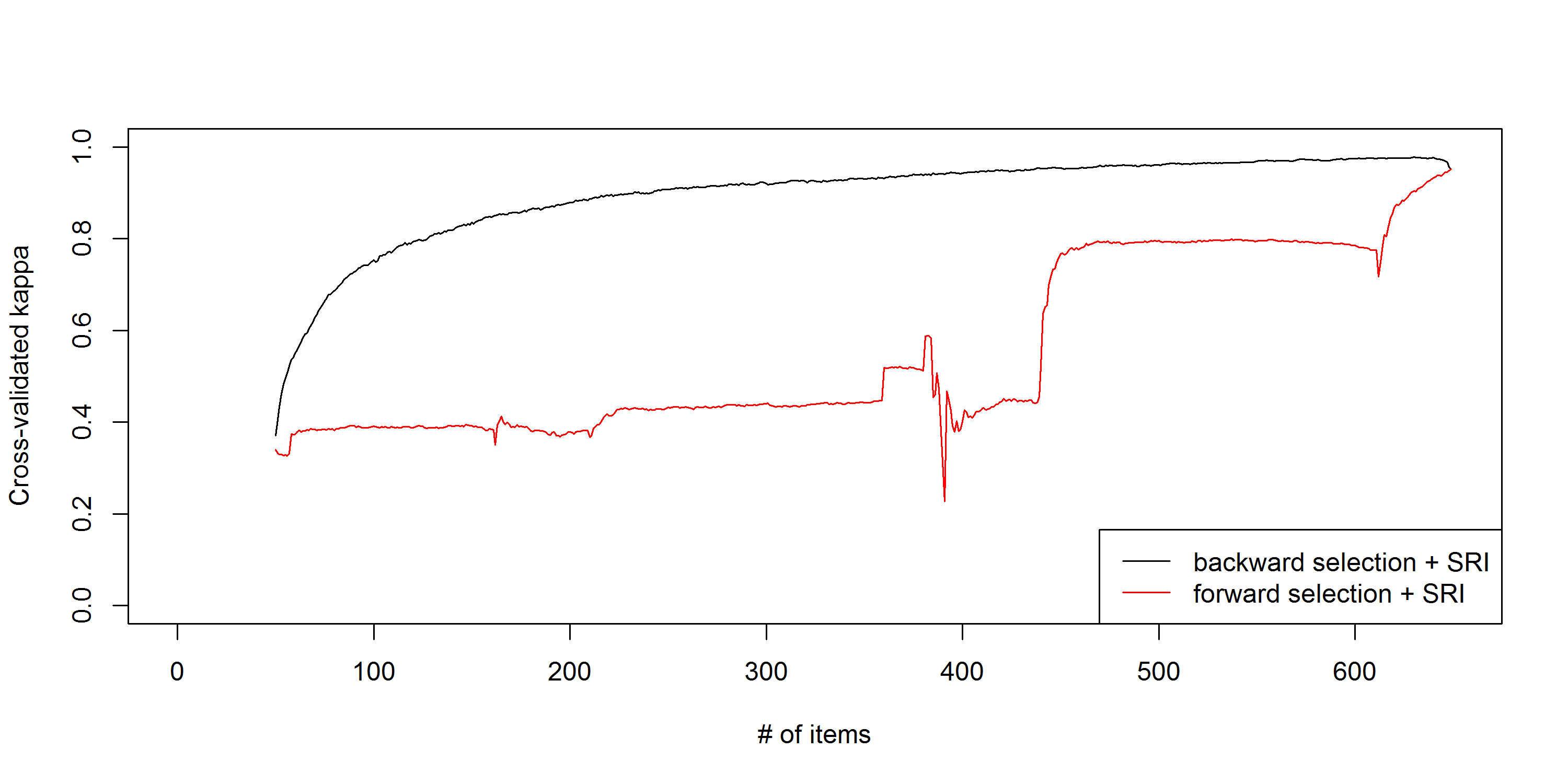

Supplement: S2 Fig — (TIFF) [file pone.0204958.s002.tiff]
