# Supplementary material for: Health system measurement: Harnessing machine learning to advance global health
Source: PLoS One. 2018 Oct 5;13(10):e0204958. doi: 10.1371/journal.pone.0204958 (PMC6173424; doi:10.1371/journal.pone.0204958)
Supplement: S1 Table — ANC: Antenatal care. DPT: Diphtheria, pertussis, tetanus. Hib: Haemophilus influenzae type B. HIV: Human immunodeficiency virus. IUCD: Intrauterine contraceptive device. NCD: Non-communicable disease. SRI: Service readiness index. STI: Sexually transmitted infection. TB: Tuberculosis. ZN: Ziehl-Neelsen. (DOCX) [file pone.0204958.s003.docx]

S1 Table: Items in the 100-item empirical index, ordered by domain and selection order

| **Domain** | **Item** | **Item is in SRI** | **Item is in 100-item enriched SRI** | **Selection order** |
| --- | --- | --- | --- | --- |
| Amenities | Sick child exam area has visual and auditory privacy | 0 | 0 | 6 |
|  | ANC exam area private room | 0 | 1 | 11 |
|  | STI consultation area has visual and auditory privacy | 0 | 0 | 14 |
|  | Facility has access to internet or email | 0 | 0 | 24 |
|  | Phlebotomy area private room | 0 | 1 | 35 |
|  | Delivery area private room | 0 | 0 | 39 |
|  | Phone or short-wave radio available at all times | 1 | 1 | 42 |
|  | Improved water source available year-round within 500 meters | 1 | 1 | 56 |
|  | TB exam area private room | 0 | 0 | 58 |
|  | Functional ambulance with fuel | 1 | 1 | 59 |
|  | Vaccination area has visual and auditory privacy | 0 | 1 | 61 |
|  | Family planning exam area private room | 0 | 0 | 62 |
|  | Facility floor is swept clean, counters/tables/chairs are wiped and clean | 0 | 0 | 76 |
|  | STI exam area private room | 0 | 0 | 85 |
|  | Family planning exam area has visual and auditory privacy | 0 | 1 | 92 |
|  | Sick child exam area: private room | 0 | 0 | 100 |
| Equipment | Blood typing: at least one valid anti-A reagent observed | 0 | 0 | 2 |
|  | Suture material with needle observed in delivery area | 0 | 1 | 7 |
|  | Filled oxygen cylinder available and functioning in outpatient area | 0 | 1 | 20 |
|  | Speculum in family planning area (if IUCDs offered) | 0 | 0 | 23 |
|  | Functional thermometer in NCD service area | 0 | 0 | 27 |
|  | Immunization tally sheets observed and in stock | 0 | 0 | 32 |
|  | Stadiometer available and functioning in outpatient area | 0 | 0 | 40 |
|  | Functional stethoscope in family planning area | 0 | 0 | 44 |
|  | Newborn mask and bag available in delivery area | 0 | 0 | 50 |
|  | Micronebulizer available and functioning in NCD service area | 0 | 0 | 57 |
|  | Functional digital or manual blood pressure apparatus in NCD service area | 0 | 0 | 70 |
|  | Bed or table in delivery area | 0 | 0 | 71 |
|  | Sterile gloves for IUCD or implant methods | 0 | 0 | 75 |
|  | Functional retractor observed in surgical area | 0 | 0 | 79 |
|  | Coldbox/vaccine carrier with ice packs observed | 0 | 0 | 82 |
|  | Functional exam light in delivery area | 0 | 0 | 87 |
|  | Chlorhexidine solution observed and valid in delivery area | 0 | 0 | 95 |
| Infection control | Blood draw area: sharps box observed | 0 | 0 | 1 |
|  | TB exam area: sharps box observed | 0 | 1 | 4 |
|  | Outpatient exam area: pourable water observed | 0 | 0 | 12 |
|  | Delivery room: single-use syringe observed | 0 | 0 | 18 |
|  | Child vaccination area: face mask observed | 0 | 1 | 25 |
|  | Outpatient exam area: hand disinfectant observed | 0 | 0 | 31 |
|  | Sick child exam room: surface disinfectant observed | 0 | 0 | 37 |
|  | ANC exam room: gloves observed | 0 | 0 | 43 |
|  | Outpatient exam area: goggles observed | 0 | 0 | 49 |
|  | Lab: gloves observed | 0 | 0 | 55 |
|  | Outpatient exam area: single-use syringe observed | 0 | 0 | 65 |
|  | Outpatient exam area: waste bin (lid and liner) observed | 0 | 0 | 66 |
|  | ANC exam room: face mask observed | 0 | 0 | 78 |
|  | Sick child exam room: soap observed | 0 | 0 | 83 |
|  | ANC exam room: surface disinfectant observed | 0 | 0 | 86 |
|  | At least one room with hand disinfectant | 0 | 0 | 90 |
|  | TB exam area: soap observed | 0 | 1 | 94 |
|  | ANC exam room: pourable water observed | 0 | 0 | 97 |
|  | ANC exam room: waste bin (lid and liner) observed | 0 | 0 | 98 |
| Diagnostics | Urine protein test observed and valid in ANC area | 0 | 0 | 5 |
|  | Functional urine dipstick test for glucose | 1 | 1 | 13 |
|  | Functional malaria testing | 1 | 1 | 19 |
|  | Functional light or fluorescent microscope and slides | 0 | 0 | 26 |
|  | HIV rapid diagnostic test observed and valid in delivery area | 0 | 0 | 28 |
|  | Functional syphilis testing | 1 | 1 | 41 |
|  | Functional blood chemistry analyzer for liver/renal function tests | 0 | 0 | 51 |
|  | Urine glucose test observed and valid in ANC area | 0 | 1 | 52 |
|  | Functional blood glucose testing | 1 | 1 | 54 |
|  | HIV rapid diagnostic test observed and valid in STI area | 0 | 1 | 68 |
|  | Diagnostics: TB test with light or fluorescent microscope, slides & ZN stain | 0 | 1 | 72 |
|  | HIV rapid diagnostic test observed and valid in HIV area | 0 | 1 | 81 |
|  | HIV rapid diagnostic test observed and valid in ANC area | 0 | 1 | 91 |
| Medication | Efavirenz tablets/capsules observed and valid | 0 | 0 | 9 |
|  | Diazepam injection (anxiety/muscle relaxant/anticonvulsant) observed and valid | 0 | 0 | 10 |
|  | DPT+Hib+HepB vaccine observed, in stock, and valid | 0 | 1 | 16 |
|  | Valid tetracycline ointment for newborn in delivery area | 0 | 0 | 22 |
|  | Isoniazid + rifampicin + pyrazinamide + ethambutol (4fdc) observed and valid | 0 | 1 | 29 |
|  | Iron (alone or with folate) observed and valid | 0 | 0 | 34 |
|  | Prednisolone observed and valid | 0 | 0 | 38 |
|  | Nevirapine syrup observed and valid | 0 | 0 | 46 |
|  | Penicillin injection (broad spectrum injectable antibiotic) | 0 | 0 | 48 |
|  | Aspirin tablets observed and valid | 0 | 0 | 60 |
|  | Progestin-only contraceptive pills observed and valid | 0 | 0 | 64 |
|  | Ketamine (injectable), observed at least 1 valid | 0 | 0 | 67 |
|  | Co-trimoxazole (tabs) (oral antibiotics-adult formation) | 0 | 0 | 77 |
|  | Ringers lactate observed and valid | 0 | 0 | 80 |
|  | Nevirapine tablets observed and valid | 0 | 0 | 88 |
|  | Beclomeasone inhaler observed and valid | 1 | 1 | 93 |
|  | Penicillin-v tablets observed and valid | 0 | 0 | 99 |
| Management | Health records for family planning clients maintained on site (observed) | 0 | 1 | 3 |
|  | Routinely carries out quality assurance activities | 0 | 1 | 8 |
|  | Health records for antenatal care clients maintained on site (observed) | 0 | 1 | 15 |
|  | Facility made decisions based on most recent management meeting | 0 | 0 | 17 |
|  | Computer or stock ledger updated daily with medicine available | 0 | 1 | 21 |
|  | Medications, vaccines and contraceptives stored according to expiry date | 0 | 1 | 30 |
|  | Supervisor discussed facility performance based on data | 0 | 1 | 33 |
|  | Health records for sick children maintained on site (observed) | 0 | 1 | 36 |
|  | Observed report of health services info compiled at least every 4-6 months | 0 | 1 | 45 |
|  | Test referral record observed in HIV or TB service | 0 | 1 | 47 |
|  | Last outside supervisory visit within 6 months | 0 | 0 | 53 |
|  | Staff community meeting within 6 months | 0 | 1 | 63 |
|  | Health records for STI clients maintained on site (observed) | 0 | 0 | 69 |
|  | Supervisor checked registers | 0 | 0 | 73 |
|  | Adequate storage for medications | 0 | 0 | 74 |
|  | Data manager responsible for health services data in this facility | 0 | 0 | 84 |
|  | Record of management team meeting observed | 0 | 1 | 89 |
|  | Record of quality assurance activities observed | 0 | 0 | 96 |

ANC: Antenatal care. DPT: Diphtheria, pertussis, tetanus. Hib: Haemophilus influenzae type B. HIV: Human immunodeficiency virus. IUCD: Intrauterine contraceptive device. NCD: Non-communicable disease. SRI: Service readiness index. STI: Sexually transmitted infection. TB: Tuberculosis. ZN: Ziehl-Neelsen.
