# Supplementary material for: Health system measurement: Harnessing machine learning to advance global health
Source: PLoS One. 2018 Oct 5;13(10):e0204958. doi: 10.1371/journal.pone.0204958 (PMC6173424; doi:10.1371/journal.pone.0204958)
Supplement: S2 Table — SRI: Service readiness index. (DOCX) [file pone.0204958.s004.docx]

S2 Table: Classification of facilities using original SRI and 100-item enriched SRI

|  |  | SRI | | | | |
| --- | --- | --- | --- | --- | --- | --- |
|  |  | Quintile 1 (Best) | Quintile 2 | Quintile 3 | Quintile 4 | Quintile 5 (Worst) |
| 100-item enriched SRI | Quintile 1, N=1848 (Best) | 71.2% | 26.0% | 2.7% | 0.1% | 0.0% |
|  | Quintile 2, N=1849 | 21.5% | 41.3% | 32.2% | 5.0% | 0.1% |
|  | Quintile 3, N=1846 | 6.2% | 21.9% | 35.7% | 30.3% | 5.9% |
|  | Quintile 4, N=1848 | 1.2% | 10.4% | 22.1% | 40.1% | 26.1% |
|  | Quintile 5, N=1847 (Worst) | 0.0% | 0.6% | 7.0% | 24.5% | 67.9% |

SRI: Service readiness index
